# Characterizing a new clinical phenotype: the co-existence of cerebral venous outflow and connective tissue disorders

**DOI:** 10.3389/fneur.2023.1305972

**Published:** 2024-01-10

**Authors:** Jackson P. Midtlien, Brian P. Curry, Emily Chang, Nicholas R. Kiritsis, Jennifer B. Aldridge, Kyle M. Fargen

**Affiliations:** Neurosurgery Department, Wake Forest School of Medicine, Winston-Salem, NC, United States

**Keywords:** Ehlers-Danlos, connective, phenotype, tissue, disorder, cerebral, idiopathic, hypermobility

## Abstract

**Background:**

There is increasing recognition of connective tissue disorders and their influence on disease in the general population. A conserved clinical phenotype involving connective tissue disorders and idiopathic intracranial hypertension (IIH) and associated cerebral venous outflow disorders (CVD) has not been previously described.

**Methods:**

A single-institution retrospective review of a prospectively maintained database of patients with connective tissue disorders and CVD was performed.

**Results:**

A total of 86 patients were identified. The majority of these patients carried a diagnosis of Ehlers-Danlos syndrome (55%) and most were non-obese (mean body mass index 29.7 kg/m2), Caucasian (90%) females (87%). Most prevalent presenting symptoms included pressure headache (98%), dizziness (90%), tinnitus (92%), and cognitive dysfunction (69%). Aside from CVD and IIH, the most common associated conditions were postural orthostatic tachycardia syndrome (POTS; 55.8%), cerebrospinal fluid (CSF) leaks (51.2%), dysautonomia (45.3%), cranio-cervical instability (37.2%), mast cell activation syndrome (25.6%), and tethered cord syndrome (23.3%). Allergies to medications (87.2%) and surgical tape (19.8%) were also frequent. Despite significantly lower opening pressures on lumbar puncture, headache severity and quality of life scores were reported with the same severity of classic IIH patients, suggesting an underlying hypersensitivity to intracranial pressures and cerebral venous congestion.

**Conclusion:**

There is a rare but conserved clinical phenotype that has not been described previously that presents with severe IIH symptoms in predominantly young, non-obese Caucasian women with a high associated incidence of dysautonomia, POTS, craniocervical instability, and CSF leaks, among others.

## Introduction

The recognition of transverse sinus stenosis as a pathophysiologic driver of intracranial venous hypertension in patients with idiopathic intracranial hypertension (IIH) has led to a dramatic shift in enthusiasm and attention surrounding IIH and its treatments (1). The frequent use of catheter venography to evaluate for stenting candidacy has resulted in a marked increase in scientific research being published on cerebral venous anatomy, physiology and mechanisms of disease (2). The increasing clinical interest and willingness to treat cerebral venous dysfunction and resulting IIH has led to an improved understanding of associated extracranial cerebral venous outflow disorders (CVD), which may be considered IIH-spectrum conditions. A number of potential causative sites of extracranial cerebral venous flow impairment have been identified (3). The most commonly identified site of outflow impairment is in the rostral internal jugular vein (IJV) near the transverse process of C1 and the styloid process, often referred to as styloidogenic jugular stenosis. Patients with symptomatic IJV stenosis present similarly to IIH patients with a predominance of complaints secondary to intracranial venous congestion including headache, skull base pain, swallowing and mastication dysfunction, brain fog, tinnitus, blurred vision and sleep disorders (4, 5). The Society of Neurointerventional Surgery recently formalized this interest in exploring CVD by creating the Cerebral Venous and Cerebrospinal Fluid Disorders Section as a means of promoting education, research, and practice guidance on these conditions (6).

Connective tissue disorders have been increasingly recognized as a cause of chronic systemic symptoms, with Ehlers-Danlos syndrome (EDS) being one of the most commonly recognized conditions. These disorders exhibit varying subtypes which have been classified previously (7); however, none of these subtypes include IIH or CVD as a notable aspect of the condition. Classically, IIH occurs in obese, child-bearing age females with high opening pressure (OP) on lumbar puncture with isolated intracranial venous sinus stenosis identified in the absence of additional extracranial sites of compression, joint hypermobility, or other systemic conditions (8). At our tertiary referral center, we have identified a conserved clinical phenotype of diagnosed or suspected connective tissue disorders and CVD in mostly young, Caucasian, non-obese women with classic symptoms and associated conditions that has not been described in the published literature. These patients are rare but have severe impairment in functional capacity and quality of life and are challenging to treat. Herein we present our series of patients to document this previously unrecognized clinical phenotype.

## Methods

A retrospective review was performed of a prospectively maintained database of patients presenting in referral at our tertiary care center for evaluation of IIH or other cerebral venous outflow disorders (CVD) with a known or highly suspected connective tissue disorder between 2019 and 2023. Patients were included based on the following criteria: (1) a diagnosis of Ehlers-Danlos syndrome (EDS) by a rheumatologist, neurologist, geneticist or other specialist; (2) a diagnosis of unspecified connective tissue disorder by a rheumatologist, neurologist, geneticist, or other specialist; (3) a suspicion of EDS or unspecified connective tissue disorder by a specialist based on associated symptoms without a formal diagnosis, with agreement by the senior author following evaluation.

Presenting symptoms were catalogued and reported (Table 1). Headache Impact Test 6 (HIT-6) and WHO-BREF Quality of Life survey responses were documented for each patient at time of first consultation. Pertinent medical history and specific diagnoses were recorded from each patient’s electronic medical record (Table 2). A diagnosis of IIH was defined based on Dandy criteria and includes at least one measured opening pressure of 25 cm of water or higher on lumbar puncture. A diagnosis of jugular stenosis was defined as (1) a history of IJV revascularization procedure including styloidectomy, open jugular decompression, or jugular stenting; or (2) findings from invasive testing performed by the senior author indicating symptomatic IJV where a jugular vein revascularization procedure was planned after testing. For all other conditions, diagnoses were not made by the authors but instead presence or absence of a diagnosis was made strictly via thorough review of patient history and previous medical records.

**Table 1:** Most common presenting symptoms.

Symptom	Prevalence (*n* = 86)
**Pressure Headache**	84 (97.7%)
**Dizziness**	77 (89.5%)
**Brain Fog/memory problems**	59 (68.7%)
**Tinnitus**	79 (91.9%)
Ringing Tinnitus	48 (55.8%)
Pulsatile Tinnitus	42 (48.8%)
**Positional change exacerbation**	42 (48.8%)
**Papilledema**	18 (20.9%)

HIT-6, Tinnitus, and WHO-BREF quality of life Scores	*N* = 76
HIT-6	65.9 (9.25)
WHO-BREF Quality of Life	171.4 (269)

**Table 2:** Prevalence of associated conditions and surgeries.

Condition	Prevalence (*n* = 86)
**Idiopathic Intracranial Hypertension (IIH)**	65 (75.6%)
History of venous sinus stenting procedure	19 (22.1%)
History of CSF shunting procedure	15 (17.4%)
**Postural Orthostatic Tachycardia Syndrome (POTS)**	48 (55.8%)
History of intravenous catheter placement for infusions	5 (5.8%)
**Internal Jugular Vein Stenosis**	45 (52.3%)
History of jugular decompression/styloidectomy procedure	27 (31.4%)
History of jugular vein stenting procedure	6 (7.0%)
**CSF leak**	44 (51.2%)
Cranial CSF leak	35 (40.7%)
Spinal CSF leak after lumbar puncture	15 (31.9%)
**Dysautonomia**	39 (45.3%)
**Joint Hypermobility**	33 (38.4%)
**Cranio-Cervical Instability (CCI)**	32 (37.2%)
**Mast Cell Activation Syndrome (MCAS)**	22 (25.6%)
**Chiari Malformation**	22 (25.6%)
History of Chiari decompression procedure	12 (14.0%)
**Tethered cord syndrome (TCS)**	20 (23.3%)
History of tethered cord release procedure	20 (23.3%)
**Obstructive Sleep Apnea (OSA)**	10 (11.6%)
**Fibromyalgia**	3 (3.5%)
**Systemic Venous Compression Syndromes**	9 (10.5%)
Nutcracker Syndrome	6 (7.0%)
May-Thurner Syndrome	4 (4.7%)
Thoracic Outlet Syndrome	1 (1.2%)

### Cerebral venogram procedural technique

All included patients underwent a diagnostic cerebral arteriogram and venogram followed by lumbar puncture for evaluating the presence of intracranial or extracranial venous stenosis, opening pressure and response to fluid drainage. Retrograde cerebral venography and manometry techniques have been described in detail previously (8, 9). In brief, diagnostic arteriography and venography was performed by a single operator, using the same procedural technique, under mild sedation (100 mcg intravenous fentanyl or less) in all patients. IIH medications are held for 48 h prior to the procedure. 5F femoral artery and vein access was obtained and following arteriography, a 5F diagnostic catheter was placed in the dominant (or co-dominant) IJV. A 0.027 inch microcatheter was navigated over a 0.014 inch microwire through the dominant transverse sinus (TS) into the superior sagittal sinus (SSS) S2 segment. The microcatheter was then connected to a pressure transducer, flushed, and then zeroed in the mid-axillary line. Mean pressures were recorded at defined locations, including S1-2 junction of the SSS, torcula, dominant TS (recorded at the lateral orbital wall on anteroposterior view), dominant SS (recorded at beginning of the horizontal segment), IJV (bulb above C1), IJV at C4 and often C6, and central venous pressure (CVP; cavo-atrial junction). For this study’s purposes, only dominant pathway pressures were included; often bilateral transverse sinus manometry is performed, however. Jugular testing is then performed in both IJV, which includes provocative testing and head rotation maneuvers to evaluate gradients in various positions. Following completion of venography, patients are then placed in the lateral decubitus position and a fluoroscopically guided lumbar puncture is performed using a 22-gauge atraumatic needle. An opening pressure (OP) is measured with a manometer and then fluid is removed to an appropriate closing pressure (usually around 10 cm of water) to document subjective symptomatic improvement with fluid removal. Procedural data for patients that underwent cerebral venography and/or lumbar puncture are presented in Table 3.

**Table 3:** Results of cerebral arteriography, venography, and LP among 86 patients.

Cerebral Arteriography	*N* = 86
Brain Aneurysms	0 (0%)
Arteriovenous Fistulae	0 (0%)
Fibromuscular Dysplasia	0 (0%)
Extracranial Arterial Dissection or Pseudoaneurysm	1 (1.2%)

Cerebral Venography	*N* = 86
Venous outflow dominance pattern	
Right	58 (67.4%)
Left	12 (14.0%)
Co-dominant	16 (18.6%)
Superior Sagittal Sinus Stenosis	
> = 4 mmHg gradient	5 (5.8%)
Dominant Transverse Sinus Stenosis	
> = 4 mmHg gradient	13 (15.1%)
> = 8 mmHg gradient	10 (11.6%)
Dominant IJ Stenosis (neutral head position)	
> = 2 mmHg gradient	10 (11.6%)
> = 4 mmHg gradient	7 (8.1%)
Non-Dominant IJ Stenosis (neutral head position)	
> = 2 mmHg gradient	14 (16.3%)
> = 4 mmHg gradient	5 (5.8%)

Lumbar Puncture	*N* = 83
Mean (IQR) Opening Pressure (cm of water)	18.5 (7)
<10 cm of water	2 (2.4%)
10–14 cm of water	22 (26.5%)
15–19 cm of water	29 (34.9%)
20–24 cm of water	18 (21.7%)
25 cm of water or higher	12 (14.5%)

### Statistical analysis

Data was analyzed using descriptive statistics. Categorical variables were described using counts and percentages, whereas continuous variables were reported using means and interquartile ranges (IQR).

## Results

### Demographics

The cohort was comprised of 86 patients, the majority of whom were female (87.2%, *n* = 75), with a mean age of 36.6 (IQR: 18) and an average BMI (Body Mass Index) of 27.9 kg/m^2^ (IQR: 10.6). Mean height and weight were 1.68 m (0.13) and 79.0 kg (30.3), respectively. Of the sample, 6.0% were underweight, 32.1% normal weight, 25.0% overweight, and 36.9% obese based on standard BMI criteria. Most patients were Caucasian (90%); a small minority were Asian (1.2%) and of African descent (2.3%). The majority of the sample included patients from the southeast region of the United States (54%), with the remainder being comprised of northeast (9.6%), Midwest (7.2%), northwest (22.0%), and southwest (7.2%).

Of the 86 patients, 48 (55.8%) had a diagnosis of EDS made previously by a rheumatologist, geneticist, neurologist or other specialist. Of the remaining patients, 3 (3.5%) had a diagnosis of unspecified connective tissue disorder and 35 (40.7%) did not have an established diagnosis but a connective tissue disorder was highly suspected by referring physicians and corroborated by the senior author.

### Presenting symptoms

Patient presenting complaints are shown in Table 1. Pressure headaches were present in most patients (97.7%), while papilledema was present in only a minority of patients (20.9%). HIT-6 scores and WHO-BREF quality of life composite scores were recorded in 76 (88.4%) of patients at the time of their first consultation and are shown in Table 1.

### Associated conditions

**Figure 1:**
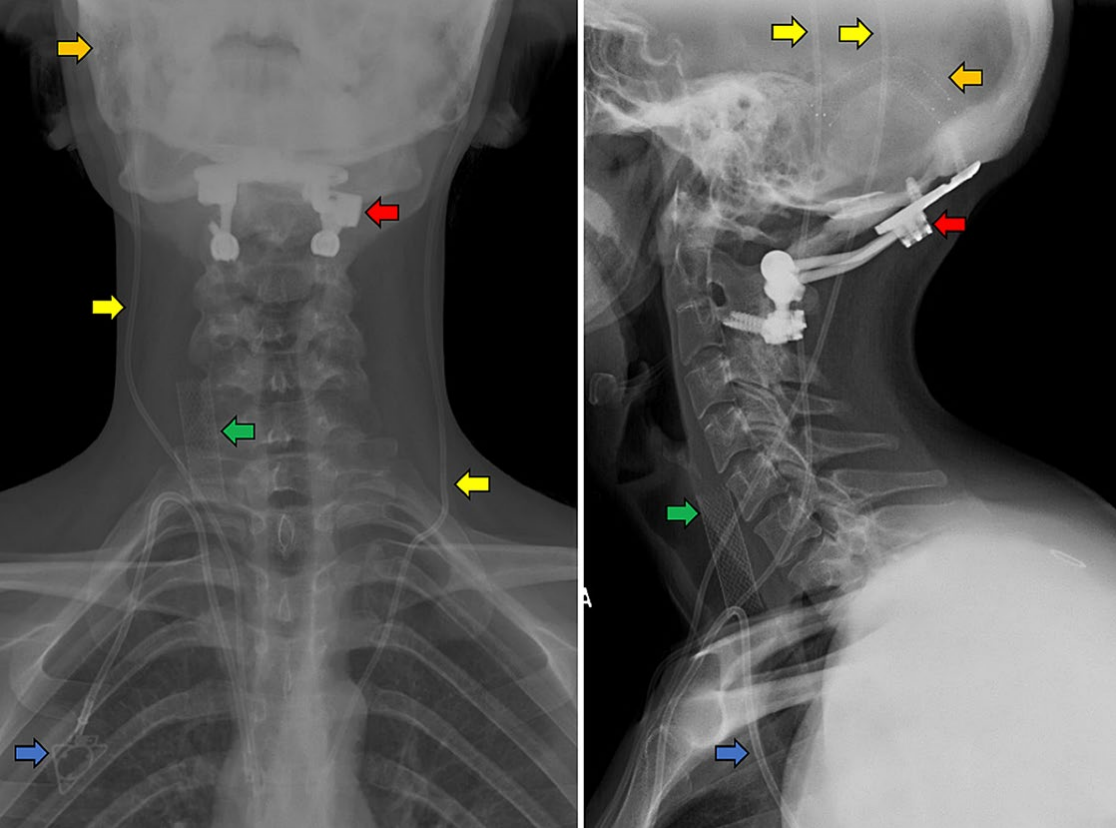
Anteroposterior (left panel) and lateral (right panel) cervical x-rays of an example patient with CVD and EDS. A Caucasian, tall, non-obese female in her 20’s with hypermobility and loose skin who has previously undergone transverse sinus stenting (orange arrows) and bilateral ventriculoperitoneal shunting (yellow) for IIH with associated CSF leaks, occipito-cervical fusion (red) for CCI, right IJV stenting (green) and port placement for fluid infusions for POTS (blue).

Table 2 lists associated conditions among the sample, in order of prevalence (Figure 1). The most common associated conditions were POTS, CSF leak, and dysautonomia. The mean number of allergies listed among the sample was 5.4 (IQR 5.5). Notably, 17 (19.8%) of patients reported adhesive tape allergies and 75 (87.2%) had allergies to at least 1 medication with a mean of 4 medication allergies among the group. 62 (72.1%) patients had previously undergone LP prior to consultation, with 15 (24.2%) patients having a history of CSF leak following LP, of which 9 required blood patching.

### Results of cerebral venography and lumbar puncture

**Figure 2:**
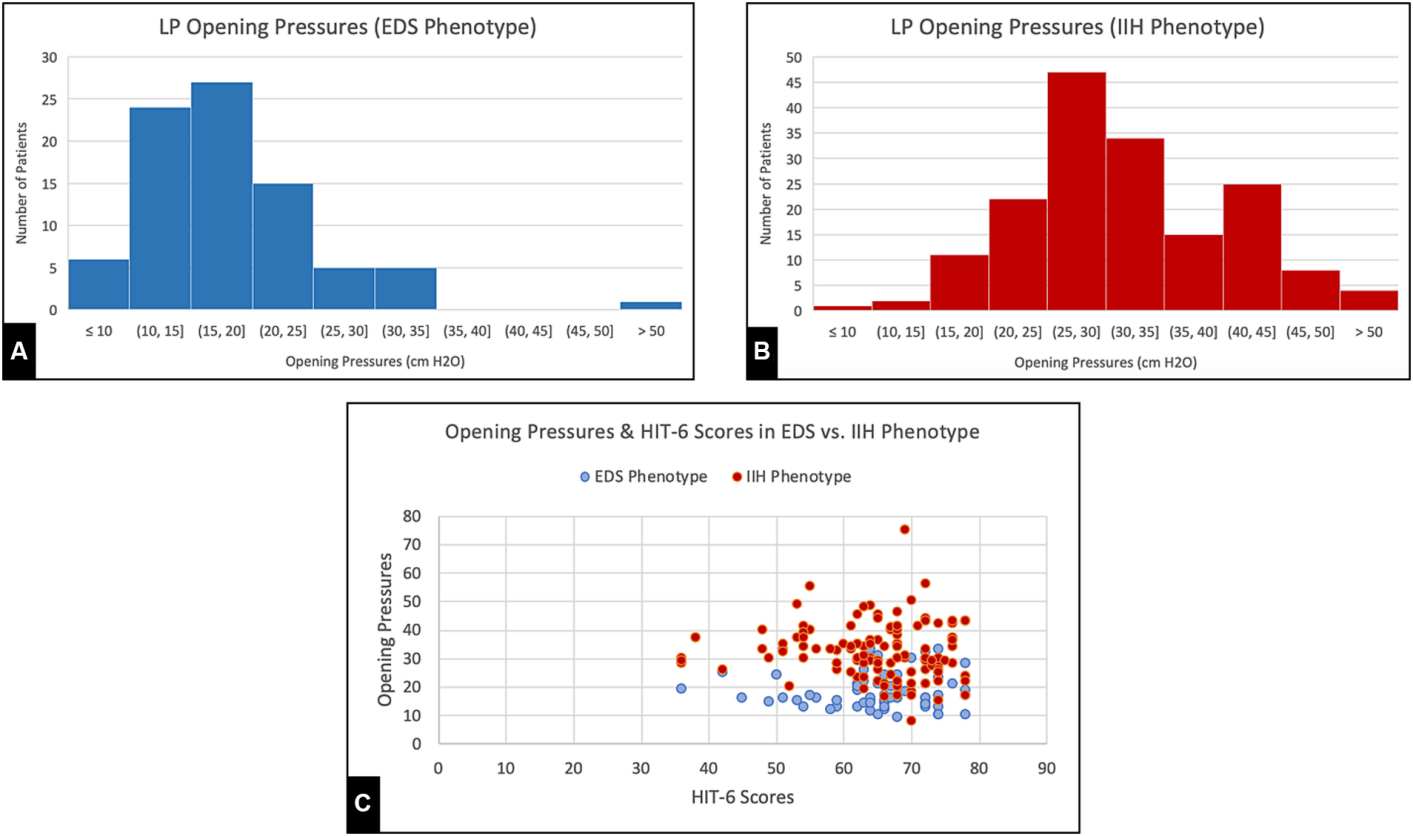
**(A,B)** Opening pressures recorded from lumbar punctures during initial visit for EDS phenotype patients and generalized IIH patients, respectively. **(C)** Headache Impact Test (HIT-6) Scores and Opening pressures collected from both EDS and generalized IIH groups.

All patients underwent cerebral arteriography, venography, and LP as part of their evaluation at our center. Findings from this study are reported in Table 3. A comparison of opening pressures and symptom severity scores between the present series and a previously reported classic sample of IIH patients is shown in Figure 2.

## Discussion

This is the first description of a clinical phenotype in patients with symptoms of CVD and have accompanying connective tissue disorders. In addition to common IIH symptoms of headaches, tinnitus, brain fog, dizziness and barometric pressure sensitivity, there is a high prevalence of conserved associated conditions including styloidogenic jugular stenosis, POTS, dysautonomia, mast cell activation syndrome, and cervical instability (CCI) in these patients. A significant portion also had a history of cerebrospinal fluid (CSF) leaks, Chiari malformations and tethered cord syndrome. Damage to the dura mater during lumbar punctures can cause cerebrospinal fluid (CSF) leaks, inducing intracranial hypotension with a range of symptoms including positional headaches. Such leaks are notably prevalent in cases involving connective tissue disorders, underscoring the need for cautious procedural and post-procedural evaluations (10).These findings contribute novel insights into the interconnectedness of these conditions and their shared symptomatology.

Many of the demographic and clinical features of this cohort demonstrate notable distinctions from the standard patient profile associated with classic IIH (11). In standard IIH, the typical profile is predominantly obese women of childbearing age of diverse racial backgrounds. The present patient sample exhibits a mean BMI much lower than previously reported in IIH and features predominantly non-obese Caucasian young women (11). Interestingly, our patients also had severe, stereotypical symptoms at relatively lower opening pressures on lumbar puncture and lower mean SSS pressures on venography compared to previous studies on general IIH patients, as compared in Figure 2 (8, 11). Moreover, headache severity (HIT-6) and quality of life (WHO-BREF) impairments were significantly worse when compared to previous analyses of IIH patients as well as the general population (11). In fact, the magnitude of patient-reported headache scores and QOL scores in this sample are notably worse than those reported for other conditions in large series (12, 13), which suggests a foundational hypersensitivity to pain or pressure dysregulation in these patients. While the physiology underlying the unique linkage between EDS and IIH is still widely unknown, abnormal levels of growth factors, such as IGF-1, have been proposed as one possible cause (14). Interestingly, such connective tissue disorders have also been linked to intracranial hypotension syndrome (IHS), with complications such as dural ectasia being common yet often under-recognized. In direct opposition to standard IIH, this association only further demonstrates the vast complexity of pathology seen in connective tissue disorders, particularly CSF pressure-dependent manifestations (15). Regardless, these findings necessitate a more nuanced understanding of CVD and accompanying connective tissue conditions and suggests these patients may have a heightened sensitivity to changes in ICP, allowing symptoms to persist at lower pressures than traditionally expected.

Connective tissue disorders, such as Ehlers-Danlos Syndrome (EDS), encompass a variety of syndromes typically characterized by joint hypermobility, skin extensibility, and tissue fragility (7). However, the manifestation of EDS varies significantly among patients and across its thirteen subtypes acknowledged by the Ehlers-Danlos Society. Our patients exhibit clinical features that align with several EDS characteristics, most notably the high prevalence of dizziness and orthostatic intolerance, often seen due to development of dysautonomia (16). However, this group also displays a unique combination of associated conditions, including MCAS, cervical instability, Chiari malformation, and tethered cord syndrome. These conditions have historically lacked concrete evidence of association within a single recognized EDS subtype, although some relations have been recently suggested (17, 18). Combined with an unusually high prevalence of jugular stenosis and IIH, these disparities only further emphasize the need to further explore the complicated pathophysiology observed in these patients (19).

The conglomerate array of clinical presentations and associated conditions in these patients makes it difficult to provide effective medical management. They tend to exhibit hypersensitivity with higher rates of all types of allergies, predominantly medication allergies. For example, a recent study found a significant incidence of atopy in pediatric populations with CVD who presented with similar phenotypic characteristics, including a lower BMI (20). Hypersensitivity, likely related to MCAS development, has been shown to be associated with EDS and POTS, and atopic conditions like eosinophilic esophagitis have been found to manifest in these patients (21). These manifestations considerably narrow the selection of safe and effective treatment modalities for these patients. For example, recent literature has suggested significant variation in surgical outcomes based on EDS subtype variation, particularly those with accompanying IIH (22). Without prior screenings and precautions, routine procedures could turn into high-risk situations, driven by undetected allergies to medications, sutures, or even adhesive tape.

In practice, the severity of these conditions can be striking, as denoted by the very high HIT-6 scores and poor WHO BREF quality of life scores. Many patients are unable to physically sit or stand and are often living with their parents or caregivers. Pain management in standard CVD and EDS patients is historically difficult, and this phenotype’s tendency towards hypermetabolism and a significant resistance to pain medications further restricts symptom management options and likely affect their quality of life (23, 24). Since hypothesized associations among these conditions and how they present is still very new and widely unrecognized, misdiagnosis continues to be a common problem in these patients (17). To help avoid misdiagnosis, improper treatment, and prolongation of symptoms, it is paramount that patients fitting this phenotype find practitioners that are knowledgeable and experienced with these associations. Clinicians evaluating patients who present similarly but out of proportion to standard CVD or connective tissue-related disorders should consider referring their them to a medical geneticist or rheumatologist (23).

Several limitations need to be considered in interpreting these findings. First, all patients were referred to and seen at a single institution, which may limit the generalizability of the findings to a broader population. The prevalence of specific conditions, such as internal jugular vein stenosis or IIH, may be influenced by referral patterns or specific patient characteristics within the population. Furthermore, our findings rely on self-reported diagnoses and medical records, which may introduce potential biases or inaccuracies. Previous diagnoses of these diseases are based on clinical assessments and may vary depending on the expertise and diagnostic criteria used. Perhaps the largest limitation remains the absence of a definitive genetic mutation that can be identified to establish a genotype associated with this unique patient cohort. As a result, current diagnosis of these conditions relies almost entirely on clinical findings rather than genetic markers. While genetic confirmation would enhance diagnostic precision, the current phenotypical characterization remains significant in advancing our understanding of these individuals. Future research should aim to integrate genetic analyses to complement the suggested phenotype, refining diagnostic accuracy and further elucidating underlying genetic factors.

## Conclusion

This study presents a novel clinical phenotype characterized by the association of connective tissue/hypermobility disorders with cerebral venous outflow disorders manifesting predominantly as symptoms of pressure headache, dizziness, tinnitus, cognitive dysfunction, barometric pressure sensitivity and visual symptoms. The overwhelming majority of patients are non-obese Caucasian women with a foundational component of hypersensitivity to CSF and venous pressures with a conserved collection of associated conditions including POTS, MCAS, CCI, Chiari malformation, CSF leaks, tethered cord and systemic venous compression syndromes. These patients endorse severe symptoms at lower intracranial pressures than expected, challenging the traditional understanding of CVD symptomatology, with markedly severe self-reported headache and quality of life scores. It is imperative that physicians recognize this distinct phenotype and are knowledgeable about the intricacies and potential complications associated with treating these conditions. A continued evolution in the understanding of this population and how they present in the clinical setting is crucial. This effort will promote the development of new interventions and enhance the integrity of the current diagnostic processes with the goal of improving the well-being and quality of life of these individuals.

## Data availability statement

The original contributions presented in the study are included in the article/supplementary material, further inquiries can be directed to the corresponding author.

## Author contributions

JM: Conceptualization, Data curation, Investigation, Methodology, Writing – original draft, Writing – review & editing, Formal analysis. BC: Conceptualization, Investigation, Methodology, Writing – original draft. EC: Data curation, Writing – review & editing. NK: Data curation, Writing – review & editing. JA: Conceptualization, Data curation, Investigation, Resources, Writing – review & editing. KF: Conceptualization, Investigation, Methodology, Project administration, Supervision, Writing – review & editing.
